# Learning-assisted theorem proving with millions of lemmas^☆^

**DOI:** 10.1016/j.jsc.2014.09.032

**Published:** 2015-07

**Authors:** Cezary Kaliszyk, Josef Urban

**Affiliations:** a University of Innsbruck, Austria; b Radboud University, Nijmegen, Netherlands

**Keywords:** Flyspeck, Lemma mining, Machine learning, Artificial intelligence

## Abstract

Large formal mathematical libraries consist of millions of atomic inference steps that give rise to a corresponding number of proved statements (lemmas). Analogously to the informal mathematical practice, only a tiny fraction of such statements is named and re-used in later proofs by formal mathematicians. In this work, we suggest and implement criteria defining the estimated usefulness of the HOL Light lemmas for proving further theorems. We use these criteria to mine the large inference graph of the lemmas in the HOL Light and Flyspeck libraries, adding up to millions of the best lemmas to the pool of statements that can be re-used in later proofs. We show that in combination with learning-based relevance filtering, such methods significantly strengthen automated theorem proving of new conjectures over large formal mathematical libraries such as Flyspeck.

## 1 Introduction: automated reasoning over large mathematical libraries

In the last decade, large formal mathematical corpora such as the Mizar Mathematical Library (Grabowski et al., 2010) (MML), Isabelle/HOL (Wenzel et al., 2008) and HOL Light (Harrison, 1996a)/Flyspeck (Hales, 2006) have been translated to formats that allow easy experiments with external automated theorem provers (ATPs) and AI systems (Urban, 2004; Meng and Paulson, 2008; Kaliszyk and Urban, 2014).

The problem that has immediately emerged is to efficiently perform automated reasoning over such large formal mathematical knowledge bases, providing as much support for authoring computer-understandable mathematics as possible. Reasoning with and over such large ITP (interactive theorem proving) libraries is however not just a new problem, but also a new opportunity, because the libraries already contain a lot of advanced knowledge in the form of concepts, theorems, proofs, and whole theory developments. Such large pre-existing knowledge allows mathematicians to state more advanced conjectures, and experiment on them with the power of existing symbolic reasoning methods. The large amount of mathematical and problem-solving knowledge contained in the libraries can be also subjected to all kinds of knowledge-extraction methods, which can later complement more exhaustive theorem-proving methods by providing domain-specific guidance. Developing the strongest possible symbolic reasoning methods that combine such knowledge extraction and re-use with correct deductive search is an exciting new area of artificial intelligence and symbolic computation.

Several symbolic AI/ATP methods for reasoning in the context of a large number of related theorems and proofs have been suggested and tried already, including: (i) methods (often external to the core ATP algorithms) that select relevant premises (facts) from the thousands of theorems available in such corpora (Meng and Paulson, 2009; Hoder and Voronkov, 2011; Kühlwein et al., 2012), (ii) methods for internal guidance of ATP systems when reasoning in the large-theory setting (Urban et al., 2011), (iii) methods that automatically evolve more and more efficient ATP strategies for the clusters of related problems from such corpora (Urban, 2014), and (iv) methods that learn which of such specialized strategies to use for a new problem (Kühlwein et al., 2013b).

In this work, we start to complement the first set of methods – ATP-external premise selection – with *lemma mining* from the large corpora. The main idea of this approach is to enrich the pool of human-defined main (top-level) theorems in the large libraries with the most useful/interesting lemmas extracted from the proofs in these libraries. Such lemmas are then eligible together with (or instead of) the main library theorems as the premises that are given to the ATPs to attack new conjectures formulated over the large libraries.

This high-level idea is straightforward, but there are a number of possible approaches involving a number of issues to be solved, starting with a reasonable definition of a *useful/interesting lemma*, and with making such definitions efficient over corpora that contain millions to billions of candidate lemmas. These issues are discussed in Sections 4 and 5, after motivating and explaining the overall approach for using lemmas in large theories in Section 2 and giving an overview of the recent related work in Section 3.

As in any AI discipline dealing with large amount of data, research in the large-theory field is driven by rigorous experimental evaluations of the proposed methods over the existing corpora. For the first experiments with lemma mining we use the HOL Light system, together with its core library and the Flyspeck library. The various evaluation scenarios are defined and discussed in Section 6, and the implemented methods are evaluated in Section 7. Section 8 discusses the various future directions and concludes.^1^

## 2 Using lemmas for theorem proving in large theories

The main task in the automated reasoning in large theories (ARLT) domain is to prove new conjectures with the knowledge of a large body of previously proved theorems and their proofs. This setting reasonably corresponds to how large ITP libraries are constructed, and hopefully also emulates how human mathematicians work more faithfully than the classical scenario of a single hard problem consisting of isolated axioms and a conjecture (Urban and Vyskočil, 2013). The pool of previously proved theorems ranges from thousands in large-theory ATP benchmarks such as MPTP2078 (Alama et al., 2014), to tens of thousands when working with the whole ITP libraries.^2^

The strongest existing ARLT systems combine variously parametrized premise-selection techniques (often based on machine learning from previous proofs (Alama et al., 2014)) with ATP systems and their strategies that are called with varied numbers of the most promising premises. These techniques can go quite far already: when using 14-fold parallelization and 30 s wall-clock time, the  system (Kaliszyk and Urban, 2014; Kaliszyk and Urban, 2013a) can today prove 47% of the 14,185^3^ Flyspeck theorems (Kaliszyk and Urban, 2013e). This is measured in a scenario^4^ in which the Flyspeck theorems are ordered *chronologically* using the loading sequence of the Flyspeck library, and presented in this order to  as conjectures. After each theorem is attempted, its human-designed HOL Light proof is fed to the 's learning components, together with the (often numerous) ATP proofs found by  itself. This means that for each Flyspeck theorem, all human-written HOL Light proofs of all previous theorems are assumed to be known, together with all their ATP proofs found already by , but nothing is known about the current conjecture and the following parts of the library (they do not exist yet).

So far, systems like  (similar systems include Sledgehammer/MaSh (Kühlwein et al., 2013a; Blanchette et al., 2011), MizAR (Urban et al., 2013; Kaliszyk and Urban, 2013c) and MaLARea (Urban et al., 2008; Kaliszyk et al., 2014)) have only used the set of *named library theorems* for proving new conjectures and thus also for the premise-selection learning. This is usually a reasonable set of theorems to start with, because the human mathematicians have years of experience with structuring the formal libraries. On the other hand, there is no guarantee that this set is in any sense optimal, both for the human mathematicians and for the ATPs. The following three observations indicate that the set of human-named theorems may be suboptimal:

*Proofs of different length:* The human-named theorems may differ considerably in the length of their proofs. The human naming is based on a number of (possibly traditional/esthetical) criteria that may sometimes have little to do with a good structuring of the library.
*Duplicate and weak theorems:* The large collaboratively-build libraries are hard to manually guard against duplications and naming of weak versions of various statements. The experiments with the MoMM system over the Mizar library (Urban, 2006) and with the recording of the Flyspeck library (Kaliszyk and Krauss, 2013) have shown that there are a number of subsumed and duplicated theorems, and that some unnamed strong lemmas are proved over and over again.
*Short alternative proofs:* The experiments with AI-assisted ATP over the Mizar and Flyspeck libraries (Alama et al., 2012; Kaliszyk and Urban, 2014) have shown that the combined AI/ATP systems may sometimes find alternative proofs that are much shorter and very different from the human proofs, again turning some “hard” named theorems into easy corollaries.

Suboptimal naming may obviously influence the performance of the current large-theory systems. If many important lemmas are omitted by the human naming, the ATPs will have to find them over and over when proving the conjectures that depend on such lemmas. On the other hand, if many similar variants of one theorem are named, the current premise-selection methods might focus too much on those variants, and fail to select the complementary theorems that are also necessary for proving a particular conjecture.^5^

To various extent, this problem might be remedied by the alternative learning/guidance methods (ii) and (iii) mentioned in Section 1: Learning of internal ATP guidance using for example Veroff's *hint technique* (Veroff, 1996), and learning of suitable ATP strategies using systems like BliStr (Urban, 2014). But these methods are so far much more experimental in the large-theory setting than premise selection.^6^ That is why we propose and explore here the following lemma-mining approach:

(1) Considering (efficiently) the detailed graph of all atomic inferences contained in the ITP libraries. Such a graph has millions of nodes for the core HOL Light corpus, and hundreds of millions of nodes for the whole Flyspeck.
(2) Defining over such large proof graphs efficient criteria that select a smaller set of the strongest and most orthogonal lemmas from the corpora.
(3) Using such lemmas together with (or instead of) the human-named theorems for proving new conjectures over the corpora.

## 3 Overview of related work and ideas

A number of ways how to measure the quality of lemmas and how to use them for further reasoning have been proposed already, particularly in the context of ATP systems and proofs. Below we summarize recent approaches and tools that initially seemed most relevant to our work.

Lemmas are an essential part of various ATP algorithms. State-of-the-art ATPs such as Vampire (Kovács and Voronkov, 2013), E (Schulz, 2002) and Prover9 (McCune, 2005–2010) implement various variants of the ANL loop (Wos et al., 1984), resulting in hundreds to billions of lemmas inferred during the prover runs. This gave rise to a number of efficient ATP indexing techniques, redundancy control techniques such as subsumption, and also fast ATP heuristics (based on weight, age, conjecture-similarity, etc.) for choosing the best lemmas for the next inferences. Several ATP methods and tools work with such ATP lemmas. Veroff's *hint technique* (Veroff, 1996) extracts the best lemmas from the proofs produced by successful Prover9 runs and uses them for directing the proof search in Prover9 on related problems. A similar lemma-extracting, generalizing and proof-guiding technique (called *E Knowledge Base – EKB*) was implemented by Schulz in E prover as a part of his PhD thesis (Schulz, 2000).

Schulz with Denzinger also developed the epcllemma (Denzinger and Schulz, 1994, 1996) tool that estimates the best lemmas in an arbitrary DAG (directed acyclic graph) of inferences. Unlike the hint-extracting/guiding methods, this tool works not just on the handful of lemmas involved in the final refutational proof, but on the typically very large number of lemmas produced during the (possibly unfinished) ATP runs. The epcllemma's criteria for selecting the next best lemma from the inference DAG are: (i) the size of the lemma's inference subgraph based at the nodes that are either axioms or already chosen (better) lemmas, and (ii) the weight of the lemma. This lemma-selection process may be run recursively, until a stopping criterion (minimal lemma quality, required number of lemmas, etc.) is reached. Our algorithm for selecting HOL Light lemmas (Section 5) is quite similar to this.

AGIntRater (Puzis et al., 2006) is a tool that computes various characteristics of the lemmas that are part of the final refutational ATP proof and aggregates them into an overall *interestingness* rating. These characteristics include: obviousness, complexity, intensity, surprisingness, adaptivity, focus, weight, and usefulness, see (Puzis et al., 2006) for details. AGIntRater so far was not directly usable on our data for various reasons (particularly the size of our graph), but we might re-use and try to efficiently implement some of its ideas later.

Pudlák (2006) has conducted experiments over several datasets with automated re-use of lemmas from many existing ATP proofs in order to find smaller proofs and also to attack unsolved problems. This is similar to the hints technique, however more automated and closer to our large-theory setting (hints have so far been successfully applied mainly in small algebraic domains). To interreduce the large number of such lemmas with respect to subsumption he used the *CSSCPA* (Sutcliffe, 2001) subsumption tool by Schulz and Sutcliffe based on the E prover. MoMM (Urban, 2006) adds a number of large-theory features to CSSCPA. It was used for (i) fast interreduction of million of lemmas extracted (generalized) from the proofs in the Mizar library, and (ii) as an early ATP-for-ITP hammer-style tool for completing proofs in Mizar with the help of the whole Mizar library. All library lemmas can be loaded, indexed and considered for each query, however the price for this breadth of coverage is that the inference process is limited to subsumption extended with Mizar-style dependent types.

AGIntRater and epcllemma use a lemma's position in the inference graph as one of the lemma's characteristics that contribute to its importance. There are also purely graph-based algorithms that try to estimate a relative importance of nodes in a graph. In particular, research of large graphs became popular with the appearance of the World Wide Web and social networks. Algorithms such as *PageRank* (Page et al., 1998) (eigenvector centrality) have today fast approximative implementations that easily scale to billions of nodes.

## 4 The proof data

We consider two corpora: the core HOL Light corpus (SVN version 179) and the Flyspeck corpus (SVN version 3437). The core HOL Light corpus contains of 2239 named theorems, while the Flyspeck corpus consists of 23,323 named theorems. The first prerequisite for implementing and running interesting lemma-finding algorithm is the extraction of the full dependency graph containing all intermediate steps (lemmas), and identification of the named top-level theorems among them.

There are three issues with the named theorems that we initially need to address. First, many theorems in HOL Light are conjunctions. It is often the case that lemmas that deal with the same constant or theory are put in the same theorem, so that they can be passed to tactics and decision procedures as a single argument rather than a list. Second, a single theorem may be given multiple names. This is especially common in case of larger formalizations like Flyspeck. Third, even if theorems are not syntactically equal they may be alpha-equivalent. HOL Light does not natively use de Bruijn indices for representing variables, i.e., two alpha-equivalent versions of the same theorems will be kept in the proof trace if they differ in variable names. Therefore the first operation we perform is to find a unique name for each separate top-level conjunct. The data sizes and processing times of this first phase can be found in Table 1.

We next look at all the available intermediate lemmas, each of them corresponding to one of the LCF-style kernel inferences done by HOL Light. The number of these lemmas when processing Flyspeck is around 1.7 billion. Here, already performing the above mentioned reduction is hard since the whole graph with the 1.7 billion HOL Light formulas can be considered big data: it fits neither in memory nor on a single hard disk. Therefore we perform the first graph reductions already when recording the proof trace.

To obtain the full inference graph for Flyspeck we run the proof-recording version of HOL Light (Kaliszyk and Krauss, 2013) patched to additionally remember all the intermediate lemmas. Obtaining such trace for Flyspeck takes 29 hours of CPU time and 56 GB of RAM on an AMD Opteron 6174 2.2 GHz Because of the memory consumption we initially consider two versions: a) de-duplicating all the intermediate lemmas within a named theorem; we call the graph obtained in this way TRACE0, and b) de-duplicating all the lemmas; which we call TRACE1. The sizes of the traces are presented in Table 2. This time and memory consumption are much lower when working only with the core HOL Light, where a further graph optimization in this step could already be possible.

**Fig. 1:**
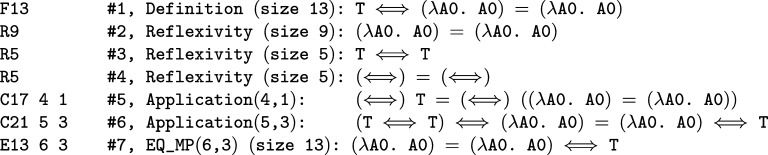
Initial segment of the HOL Light theorem trace commented with the numbers of the steps and the theorems derived by the steps.

There are 1,953,406,411 inference edges between the unique Flyspeck lemmas. During the proof recording we additionally export the information about the symbol weight (size) of each lemma (the weight is used in some of the lemma-quality metrics defined in Section 5), and for the small HOL Light traces also the lemma's normalized form that serially numbers bound and free variables and tags them with their types. This information is later used for external postprocessing, together with the information about which theorems where originally named. The initial segment of the Flyspeck proof trace is presented in Fig. 1, all the traces are available online.^7^

### 4.1 Initial post-processing and optimization of the inference traces

During the proof recording, only exact duplicates are easy to detect. As already explained in the previous section, HOL Light does not natively use de Bruijn indices for representing variables, so the trace may still contain alpha-equivalent versions of the same theorems. Checking for alpha equivalence during the proof recording would be possible, however is not obvious since in the HOL Light's LCF-style approach alpha conversion itself results in multiple kernel inferences. In order to avoid performing term-level renamings we keep the original proof trace untouched, and implement its further optimizations as external postprocessing of the trace.

In particular, to merge alpha-equivalent lemmas in a proof trace *T*, we just use the above mentioned normalized-variable representation of the lemmas as an input to an external program that produces a new version of the proof trace $T^{\prime }$. This program goes through the trace *T* and replaces references to each lemma by a reference to the earliest lemma in *T* with the same normalized-variable representation. The proofs of the later named alpha variants of the lemmas in *T* are however still kept in the new trace $T^{\prime }$, because such proofs are important when computing the usage and dependency statistics over the normalized lemmas. We have done this postprocessing only for the core HOL Light lemmas, because printing out of the variable-normalized version of the 150,142,900 partially de-duplicated Flyspeck lemmas is currently not feasible on our hardware. From the 2,076,682 partially de-duplicated core HOL Light lemmas 1,076,995 are left after this stronger normalization. We call such further post-processed graph TRACE2.

It is clear that such post-processing operations can be implemented in various ways. In this case, some original information about the proof graph is lost, while some information (proofs of duplicate lemmas) is still kept, even though it could be also pruned from the graph, producing a differently normalized version.

### 4.2 Obtaining shorter traces from the tactic calls

Considering the HOL kernel proof steps as the atomic steps in construction of intermediate lemmas has (at least) three drawbacks. First, the pure size of the proof traces makes it hard to scale the lemma-mining procedures to big developments like Flyspeck. Second, the multitude of steps that arise when applying simple HOL Light decision procedures overshadows the interesting parts of the proofs. It is not uncommon for a simple operation, like a normalization of a polynomial, to produce tens of thousands of core kernel inferences. Third, some operations (most notably the HOL Light simplifier) produce kernel inferences in the process of proof search. Such inferences are not only uninteresting (as in the previous case), but often useless for the final proof.

In order to overcome the above three issues encountered in the first experiments, we followed by gathering data at the level of the HOL Light *tactic* steps (Harrison, 1996a). The execution of each HOL Light tactic produces a new goal state together with a justification function that produces an intermediate lemma. In this approach we consider only the lemmas produced by the justification functions of tactics (instead of considering all kernel steps). The HOL Light tactics work on different levels. The tactics executed by the user and visible in the proof script form the outermost layer. However most of the tactics are implemented as OCaml functions that inspect the goal and execute other (smaller) tactics. If we unfold such internal executions of tactics recursively, the steps performed are of a similar level of detail as in typical natural deduction proofs.

This could give us a trace that is slightly smaller than the typical trace of the kernel inferences; however the size is still of the same order of magnitude. In order to efficiently process large formal developments we decided to look at an intermediate level: only at the tactics that are composed using *tactic combinators* (Harrison, 1996a).

In order to patch the tactic combinators present in HOL Light and Flyspeck it is enough to patch the three building blocks of tactic combinators: THEN, THENL, and by. Loading Flyspeck with these functions patched takes about 25% more time than the original and requires 6 GB of memory to remember all the 20 million new intermediate theorems. This is significantly less than the patched kernel version and the produced graph can be reasonably optimized.

The optimizations performed on the level of named theorems can be done here again: recursively splitting conjunctions and normalizing the quantifiers, as well as the premises we get 2,014,505 distinct conjuncts. After alpha-normalization this leaves a trace with 1,067,107 potential intermediate lemmas. In order to find dependencies between the potential intermediate lemmas we follow the approach by Kaliszyk and Krauss (2013) which needs a second dependency recording pass over the whole Flyspeck.

**Fig. 2:**
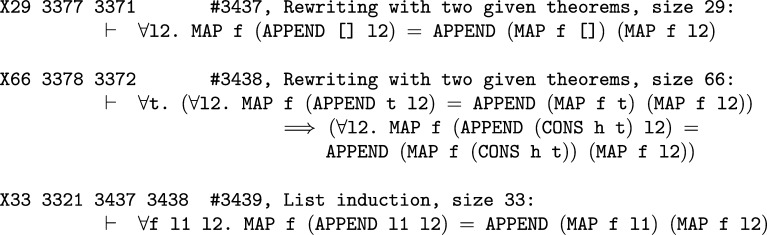
An excerpt of the tactical trace showing the dependencies between the goal states in the proof of MAP_APPEND. For simplicity we chose an excerpt that shows the theorems created by the direct application of a tactic that does not call other tactics (LIST_INDUCT_TAC). This means that all the theorems created in this part of the trace directly correspond to goals visible to the proof-assistant user.

The post-processed tactics dependency graph has 4,268,428 edges and only 2145 nodes have no dependencies. The comparison of all the traces can be seen in Table 2. The data is written in the same format as the HOL kernel inference data, so that we can use the same predictors. An excerpt from the tactical trace coming from the proof of MAP_APPEND is presented in Fig. 2.

### 4.3 Other possible optimizations

The ATP experiments described below use only the four versions of the proof trace (TRACE0, TRACE1, TRACE2, and the tactical trace) described above, but we have also explored some other normalizations. A particularly interesting optimization from the ATP point of view is the removal of subsumed lemmas. An initial measurement with the (slightly modified) MoMM system done on the classified first-order versions of about 200,000 core HOL Light lemmas has shown that about 33% of the clauses generated from the lemmas are subsumed. But again, ATP operations like subsumption interact with the level of inferences recorded by the HOL Light kernel in nontrivial ways. It is an interesting task to define exactly how the original proof graph should be transformed with respect to such operations, and how to perform such proof graph transformations efficiently over the whole Flyspeck.

## 5 Selecting good lemmas

Several approaches to defining the notion of a useful/interesting lemma are mentioned in Section 3. There are a number of ideas that can be explored and combined together in various ways, but the more complex methods (such as those used by AGIntRater) are not yet directly usable on the large ITP datasets that we have. So far, we have experimented mainly with the following techniques:

(1) A direct OCaml implementation of lemma quality metrics based on the HOL Light proof-recording data structures.
(2) Schulz's epcllemma and our modified versions thereof.
(3) PageRank, applied in various ways to the proof trace.
(4) Graph cutting algorithms with modified weighting function.

### 5.1 Direct computation of lemma quality

The advantage of the direct OCaml implementation is that no export to external tools is necessary and all the information collected about the lemmas by the HOL Light proof recording is directly available. The basic factors that we use so far for defining the quality of a lemma *i* are its: (i) set of direct proof dependencies d(i) given by the proof trace, (ii) number of recursive dependencies D(i), (iii) number of recursive uses U(i), and (iv) number of HOL symbols (HOL weight) S(i). When recursively defining U(i) and D(i) we assume that in general some lemmas may already be named (k∈Named) and some lemmas are just axioms (k∈Axioms). Note that in HOL Light there are many lemmas that have no dependencies, but formally they are still derived using for example the reflexivity inference rule (i.e., we do not count them among the HOL Light axioms). The recursion when defining *D* thus stops at axioms, named lemmas, and lemmas with no dependencies. The recursion when defining *U* stops at named lemmas and unused lemmas. Formally:

Definition 1 **Recursive dependencies and uses**

$$D(i)=\left\{ \begin{array}{cc} 1 & \text{if }i\in \text{Named}\vee i\in \text{Axioms},\\ \sum_{j\in d(i)}D(j) & \text{otherwise}, \end{array}\right.\;U(i)=\left\{ \begin{array}{cc} 1 & \text{if }i\in \text{Named},\\ \sum_{i\in d(j)}U(j) & \text{otherwise}. \end{array}\right.$$

In particular, this means that

D(i)=0⇔d(i)=∅∧¬(i∈Axioms)

and also that

U(i)=0⇔∀j¬(i∈d(j))

These basic characteristics are combined into the following lemma quality metrics $Q_{1}(i)$, $Q_{2}(i)$, and $Q_{3}(i)$. $Q_{1}^{r}(i)$ is a generalized version of $Q_{1}(i)$, which we (apart from $Q_{1}$) test for r∈{0,0.5,1.5,2}:

Definition 2 **Lemma quality**

$$Q_{1}(i)=\frac{U(i)⁎D(i)}{S(i)},\;Q_{2}(i)=\frac{U(i)⁎D(i)}{S{\left(i\right)}^{2}},$$

$$Q_{1}^{r}(i)=\frac{U{\left(i\right)}^{r}⁎D{\left(i\right)}^{2-r}}{S(i)},\;Q_{3}(i)=\frac{U(i)⁎D(i)}{1.1^{S(i)}}$$

The justification behind these definitions are the following heuristics:

(1) The higher D(i) is, the more necessary it is to remember the lemma *i*, because it will be harder to infer with an ATP when needed.
(2) The higher U(i) is, the more useful the lemma *i* is for proving other desired conjectures.
(3) The higher S(i) is, the more complicated the lemma *i* is in comparison to other lemmas. In particular, doubled size may often mean in HOL Light that *i* is just a conjunction of two other lemmas.^8^

### 5.2 Lemma quality via epcllemma

Lemma quality in epcllemma is defined on clause inferences recorded using E's native PCL protocol. The lemma quality computation also takes into account the lemmas that have been already named, and with minor implementational variations it can be expressed using *D* and *S* as follows:

$$EQ_{1}(i)=\frac{D(i)}{S(i)}$$

The difference to $Q_{1}(i)$ is that U(i) is not used, i.e., only the cumulative effort needed to prove the lemma counts, together with its size (this is also very close to $Q_{1}^{r}(i)$ with r=0). The main advantage of using epcllemma is its fast and robust implementation using the E code base. This allowed us to load in reasonable time (about one hour) the whole Flyspeck proof trace into epcllemma, taking 67 GB of RAM. Unfortunately, this experiment showed that epcllemma assumes that *D* is always an integer. This is likely not a problem for epcllemma's typical use, but on the Flyspeck graph this quickly leads to integer overflows and wrong results. To a smaller extent this shows already on the core HOL Light proof graph. A simple way how to prevent the overflows was to modify epcllemma to use instead of *D* the longest chain of inferences *L*:

$$L(i)=\left\{ \begin{array}{cc} 1 & \text{if }i\in \text{Named}\vee i\in \text{Axioms},\\ \max_{j\in d(i)}(1+L(j)) & \text{otherwise} \end{array}\right.$$

This leads to:

$$EQ_{2}(i)=\frac{L(i)}{S(i)}$$

Apart from this modification, only minor changes were needed to make epcllemma work on the HOL Light data. The proof trace was expressed as a PCL proof (renaming the HOL inferences into E inferences), and TPTP clauses were used instead of the original HOL clauses. We additionally compared two strategies of creating the TPTP clauses. First we applied the MESON (Harrison, 1996b) translation to the HOL clause, second we tried to create artificial TPTP clauses of the size corresponding to the size of the HOL clause.

### 5.3 Lemma quality via PageRank

PageRank (eigenvector centrality of a graph) is a method that assigns weights to the nodes in an arbitrary directed graph (not just DAG) based on the weights of the neighboring nodes (“incoming links”). In more detail, the weights are computed as the dominant eigenvector of the following set of equations:

$$PR_{1}(i)=\frac{1-f}{N}+f\sum_{i\in d(j)}\frac{PR_{1}(j)}{\left|d(j)\right|}$$

where *N* is the total number of nodes and *f* is a damping factor, typically set to 0.85. The advantage of using PageRank is that there are fast approximative implementations that can process the whole Flyspeck proof graph in about 10 minutes using about 21 GB RAM, and the weights of all nodes are computed simultaneously in this time.

This is however also a disadvantage in comparison to the previous algorithms: PageRank does not take into account the lemmas that have already been selected (named). The closer a lemma *i* is to an important lemma *j*, the more important *i* will be. Modifications that use the initial PageRank scores for more advanced clustering exist (Avrachenkov et al., 2008) and perhaps could be used to mitigate this problem while still keeping the overall processing reasonably fast. Another disadvantage of PageRank is its ignorance of the lemma size, which results in greater weights for the large conjunctions that are used quite often in HOL Light. $PR_{2}$ tries to counter that:

$$PR_{2}(i)=\frac{PR_{1}(i)}{S(i)}$$

$PR_{1}$ and $PR_{2}$ are based on the idea that a lemma is important if it is needed to prove many other important lemmas. This can be again turned around: we can define that a lemma is important if it depends on many important lemmas. This is equivalent to computing the reverse PageRank and its size-normalized version:

$$PR_{3}(i)=\frac{1-f}{N}+f\sum_{i\in u(j)}\frac{PR_{3}(j)}{\left|u(j)\right|},\;PR_{4}(i)=\frac{PR_{3}(i)}{S(i)}$$

where u(j) are the direct uses of the lemma *j*, i.e., i∈u(j)⇔j∈d(i). The two ideas can again be combined (note that the sum of the PageRanks of all nodes is always 1):

$$PR_{5}(i)=PR_{1}(i)+PR_{3}(i),\;PR_{6}(i)=\frac{PR_{1}(i)+PR_{3}(i)}{S(i)}$$

### 5.4 Lemma quality using graph cut

The approaches so far tried to define what a “good” lemma is using our intuitions coming from mathematics. Here we will try to estimate the impact that choosing certain lemmas will have on the final dependency graph used for the learning framework.

Choosing a subset of the potential intermediate lemmas can be considered a variant of the graph-theoretic problems of finding a cut with certain properties. We will consider only cuts that respect the chronological order of theorems in the library. Since many of the graph-cut algorithms (for example maximum cut) are NP-complete, we decide to build the cut greedily adding nodes to the cut one by one.

**Fig. 3:**
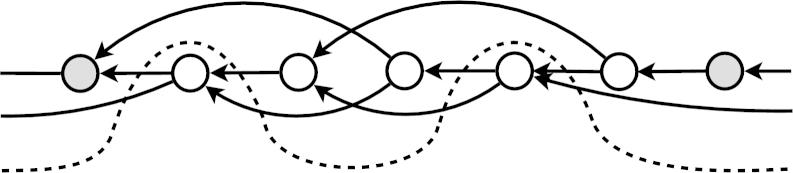
An example cut of the dependency graph that respects the chronological order of the library. The already named theorems are marked in gray.

Given a graph where certain nodes are already named (marked gray in the Fig. 3) we want to estimate the impact of choosing a new lemma on the evaluation. In the evaluation, we will compute the dependency graph of all the gray nodes together with the newly chosen one. The final graph represents the human dependencies, which means that theorems are ATP-provable using exactly these dependencies. By minimizing the number of edges in this final graph we make the average number of premises in the problems smaller which should make the problems easier to prove. The assumption here, is that training our premise-selection systems on theorems that are easier to prove makes the resulting AI/ATP system stronger.

In order to minimize the number of edges in the final graph we will investigate what is the impact of adding a node *n* to the cut. We consider all the dependency paths starting which start at *n*. On each path we select the first already named node. All the nodes that have been selected are the dependencies that *n* will have in the final dependency graph. Lets denote this set as D(n). Similarly we can find all the nodes that will have *n* as a dependency. This can be done in a similar way, taking all the paths the opposite direction again choosing the first gray node on each path. Lets denote the nodes found as U(n). These nodes will have *n* as a dependency if *n* is chosen to be in the cut.

Theorem 3 *Adding a node n to the cut c will decrease the number of edges in the final graph by* |D(n)|⁎|U(n)|−|D(n)|−|U(n)|*.*

Proof With the cut *c* the edges in the final graph include all the edges between the nodes in D(n) and U(n). Adding the node *n* to *c* these |D(n)|⁎|U(n)| edges will be replaced by the dependencies from each element of U(n) to *n* (|U(n)| many of them) and the dependencies from *n* to all the elements of D(n) (|D(n)| many of them).

The algorithm manipulates sets of nodes rather than numbers, which makes it significantly slower than all the previously described ones. We will test this algorithm only for up to 10,000 lemmas as already finding them takes 11 CPU hours. Similarly to the algorithms in the previous subsections we try to limit the effect of large theorems on the algorithm by considering also the size normalized version:

$$MC_{1}(i)=\left|D(i)⁎U(i)\right|-\left|D(i)\right|-\left|U(i)\right|,\;MC_{2}(i)=\frac{MC_{1}(i)}{S(i)}$$

### 5.5 Selecting many lemmas

From the methods described above, only the various variants of PageRank ($PR_{i}$) produce the final ranking of all lemmas in one run. Both epcllemma ($EQ_{i}$) and our custom methods ($Q_{i}$, $MC_{i}$) are parametrized by the set of lemmas (*Named*) that have already been named. When the task is to choose a predefined number of the best lemmas, this naturally leads to the recursive lemma-selection Algorithm 1 (used also by epcllemma). This algorithm in each iteration selects the lemma with the highest quality wrt. the current set of named lemmas, and adds this lemma to that set, usually making some recomputing of the lemma quality necessary in the next iteration.

There are two possible choices of the initial set of named lemmas ${\text{Named}}_{0}$ in Algorithm 1: either the empty set, or the set of all human-named theorems. This choice depends on whether we want to re-organize the library from scratch, or whether we just want to select good lemmas that complement the human-named theorems. Below we experiment with both approaches. Note that this algorithm is currently quite expensive: the fast epcllemma implementation takes 65 seconds to update the lemma qualities over the whole Flyspeck graph after each change of the *Named* set. This means that with the kernel-based inference trace (TRACE1) producing the first 10,000 Flyspeck lemmas takes 180 CPU hours. That is why most of the experiments are limited to the core HOL Light graph and Flyspeck tactical graph where this takes about 1 second and 3 hours respectively.

## 6 Evaluation scenarios and issues

To assess and develop the lemma-mining methods we define several evaluation scenarios that vary in speed, informativeness and rigor. The simplest and least rigorous is the *expert-evaluation* scenario: We can use our knowledge of the formal corpora to quickly see if the top-ranked lemmas produced by a particular method look plausible.

The *cheating ATP* scenario uses the full proof graph of a corpus to compute the set of the (typically 10,000) best lemmas (*BestLemmas*) for the whole corpus. Then the set of newly named theorems (*NewThms*) is defined as the union of *BestLemmas* with the set of originally named theorems (*OrigThms*): NewThms:=BestLemmas∪OrigThms. The derived graph $G_{\text{NewThms}}$ of direct dependencies among the elements of *NewThms* is used for ATP evaluation, which may be done in two ways: with human selection and with AI selection. When using human selection, we try to prove each lemma from its parents in $G_{\text{NewThms}}$. When using AI selection, we use the chronological order (see Section 2) of *NewThms* to incrementally train and evaluate the *k*-NN machine learner (Kaliszyk and Urban, 2013e) on the direct dependencies from $G_{\text{NewThms}}$. This produces for each new theorem an ATP problem with premises advised by the learner trained on the $G_{\text{NewThms}}$ dependencies of the preceding new theorems. This scenario may do a lot of cheating, because when measuring the ATP success on *OrigThms*, a particular theorem *i* might be proved with the use of lemmas from *NewThms* that have been stated for the first time only in the original proof of *i* (we call such lemmas *directly preceding*). In other words, such lemmas did not exist before the original proof of *i* was started, so they could not possibly be suggested by lemma-quality metrics for proving *i*. Such directly preceding lemmas could also be very close to *i*, and thus equally hard to prove.

The *almost-honest ATP* scenario does not allow the use of the directly preceding new lemmas. The dependencies of each i∈NewThms may consist only of the previous *OrigThms* and the lemmas that precede them. Directly preceding new lemmas are replaced by their closest *OrigThms* ancestors. This scenario is still not fully honest, because the lemmas are computed according to their lemma quality measured on the full proof graph. In particular, when proving an early theorem *i* from *OrigThms*, the newly used parents of *i* are lemmas whose quality was clear only after taking into account the theorems that were proved later than *i*. These theorems and their proofs however did not exist at the time of proving *i*. Still, we consider this scenario sufficiently honest for most of the ATP evaluations done with the whole core HOL Light dataset and the representative subset of the Flyspeck dataset.

The *fully-honest ATP* scenario removes this last objection, at the price of using considerably more resources for a single evaluation. For each originally named theorem *j* we limit the proof graph used for computing *BestLemmas* to the proofs that preceded *j*. Since computing *BestLemmas* for the whole core HOL Light takes at least three hours for the $Q_{i}$ and $EQ_{i}$ methods, the full evaluation on all 1954 core HOL Light theorems would take about 2000 CPU hours. That is why we further scale down this evaluation by doing it only for every tenth theorem in core HOL Light.

The *chained-conjecturing ATP* scenario is similar to the cheating scenario, but with limits imposed on the directly preceding lemmas. In ${\mathrm{chain}}_{1}$-*conjecturing*, any (possibly directly preceding) lemma used to prove a theorem *i* must itself have an ATP proof using only *OrigThms*. In other words, it is allowed to guess good lemmas that still do not exist, but such lemmas must not be hard to prove from *OrigThms*. Analogously for ${\mathrm{chain}}_{2}$-*conjecturing* (resp. ${\mathrm{chain}}_{N}$), where lemmas provable from ${\mathrm{chain}}_{1}$-lemmas (resp. ${\mathrm{chain}}_{N-1}$) are allowed to be guessed. To some extent, this scenario measures the theoretical ATP improvement obtainable with guessing of good intermediate lemmas.

## 7 Experiments

In total, we have performed experiments with 180 different strategies for adding new lemmas based on the kernel inference traces, and with 164 different strategies for adding new lemmas based on the tactical traces. The ATP experiments are done on the same hardware and using the same setup that was used for the earlier evaluations described in (Kaliszyk and Urban 2014, 2013e): All ATP systems are run with 30 s time limit on a 48-core server with AMD Opteron 6174 2.2 GHz CPUs, 320 GB RAM, and 0.5 MB L2 cache per CPU.

In order to find the exact HOL formulas corresponding to the new lemmas (known only as nodes in a graph) coming from mining the kernel inference traces, we first have to process the formalization again with a patched kernel that takes the lemma numbers as a parameter and exports also the statements of the selected new lemmas. This is no longer necessary for the tactic data, since the formula statements can be stored together with the proof graph during the first run. The slowest part of our setup is computing the formula features needed for the machine learning. For the experiments with the kernel inference lemmas, the features of each final set of selected lemmas (*NewThms*) are computed independently, since we cannot pre-compute the features of all the lemmas in the kernel traces. In case of the Flyspeck tactical trace we can directly compute the features of all of the over 1 million lemmas. Due to their size (the intermediate lemmas are often large implications), it takes 28 hours to extract and normalize (Kaliszyk and Urban, 2014) all the features. The sum of the counts of such features over all these lemmas is 63,433,070, but there are just 383,304 unique features in these lemmas. Even for the extreme case of directly using and predicting premises for all the lemmas from the Flyspeck tactical trace without any preselection, our *k*-NN predictor can perform all the one million predictions in about 30 hours, taking 0.11 s per prediction. Predictions are translated from the HOL logic into FOF problems (Kaliszyk and Urban, 2014) and ATPs are run on them in the usual way to make the evaluations.

In order to compare the new results with the extensive experimental results obtained over the previous versions of HOL Light and Flyspeck used in (Kaliszyk and Urban, 2014), we first detect the set of theorems that are preserved between the different versions. This is done by using the recursive content-based naming of symbols and theorems that we have developed for re-using as much information between different library versions in the  online service Kaliszyk and Urban (2014). In case of HOL Light the complete set of 1954 core HOL Light theorems evaluated in previous evaluations of  has been preserved, only some of the names have been changed. In case of Flyspeck a smaller set of 10,779 theorems is preserved. In order to perform more experiments we further reduced the size of this set by choosing only every sixth theorem and evaluating the performance on the resulting 1796 theorems.

### 7.1 Evaluation on core HOL Light

When using only the original theorems, the success rate of the 14 most complementary AI/ATP methods developed in (Kaliszyk and Urban, 2014) run with 30 s time limit each and restricted to the 1954 core HOL Light theorems is 63.1% (1232 theorems) and the union of all those methods solved 65.4% (1278 theorems). In the very optimistic *cheating* scenario (limited only to the $Q_{i}$ metrics), these numbers go up to 76.5% (1496 theorems) resp. 77.9% (1523 theorems). As mentioned in Section 6, many proofs in this scenario may however be too simple because a close directly preceding lemma was used by the lemma-mining/machine-learning/ATP stack. This became easy to see already when using the *almost-honest* scenario, where the 14 best methods (including also $EQ_{i}$ and $PR_{i}$) solve together only 66.2% (1293 theorems) and the union of all methods solves 68.9% (1347 theorems). The performance of the various (almost-honest) new lemma-based methods is shown in Table 3, together with their comparison and combination with the old experiments.

The majority of the new solved problems come from the alpha-normalized TRACE2, however the non-alpha normalized versions with and without duplicates do contribute as well. When it comes to the number of theorems added, adding more theorems seems to help significantly, see Table 4. We do not try to add more than 10,000 theorems for core HOL Light, as this is already much bigger than the size of the library. We will add up to one million theorems when looking at the whole Flyspeck in the next subsection.

For each of the strategies the success rates again depend on the different arguments that the strategy supports. In case of direct lemma computation considering $Q_{1}$ seems to give the best results, followed by $Q_{2}$ and $Q_{3}^{1.1}$; see Table 5. This suggest that focusing on either *U* or *D* is worse than looking at the combination. For core HOL Light size seems not to be an issue and dividing by size gives us best results. This will change in Flyspeck where the real arithmetic decision procedures produce much bigger intermediate lemmas.

In case of epcllemma three main strategies of creating a FOF trace from an inference trace were considered. First, we tried to apply the MESON translation of formulas. On one hand this was most computationally expensive as it involves lambda-lifting and introducing the apply functor, on the other hand it produces first-order formulas whose semantics are closest to those of the higher-order formulas involved. Second, we tried to create arbitrary FOF formulas of the same size as the one of the input HOL formula. Third, we modified the second approach to also initialize epcllemma with the already named theorems. The results can be found in Table 6. The size of theorems is much more important than the structure and initialization does not seem to help.

We next compare the versions of PageRank. The intersection between the first 10,000 lemmas advised by $PR_{1}$ and $PR_{2}$ is 79%, which suggests that the lemmas suggested by $PR_{1}$ are already rather small. For the reverse PageRank it is the opposite: $PR_{3}$ and $PR_{4}$ have only 11% intersection. This makes the bigger lemmas suggested by $PR_{3}$ come out second after the normalized combined $PR_{6}$ in Table 7.

The resource-intensive *fully-honest* evaluation is limited to a relatively small subset of the core HOL Light theorems, however it confirms the *almost-honest* results. While the original success rate was 61.7% (less than 14 methods are needed to reach it), the success rate with lemma mining went up to 64.8% (again, less than 14 methods are needed). This means that the non-cheating lemma-mining approaches so far improve the overall performance of the AI/ATP methods over core HOL Light by about 5%. The best method in the *fully-honest* evaluation is $Q_{2}$ which solves 46.2% of the original problems when using 512 premises, followed by $EQ_{2}$ (using the longest inference chain instead of *D*), which solves 44.6 problems also with 512 premises. The best PageRank-based method is $PR_{2}$ (PageRank divided by size), solving 41.4% problems with 128 premises.

An interesting middle-way between the cheating and non-cheating scenarios is the *chained-conjecturing* evaluation, which indicates the possible improvement when guessing good lemmas that are “in the middle” of long proofs. Since this is also quite expensive, only the best lemma-mining method ($Q_{2}$) was evaluated on the HOL Light TRACE2. $Q_{2}$ itself solves (altogether, using different numbers of premises) 54.5% (1066) of the problems. This goes up to 61.4% (1200 theorems) when using only ${\mathrm{chain}}_{1}$-*conjecturing* and to 63.8% (1247 theorems) when allowing also ${\mathrm{chain}}_{2}$ and ${\mathrm{chain}}_{3}$-*conjecturing*. These are 12.6% and 17.0% improvements respectively, see Table 8.

### 7.2 Evaluation on Flyspeck

For the whole Flyspeck the evaluation is due to the sizes of the data limited to the tactical trace and the almost-honest scenario. Table 9 (the Flyspeck counterpart of Table 3) presents the performance of the various lemma-based methods on the 1796 selected Flyspeck theorems, together with the comparison and combination with the old experiments. The combination of the 14 best methods tested here solves 37.6% problems, and the combination of all methods solves 40.8% problems. When combined with the most useful old methods developed in (Kaliszyk and Urban, 2014), the performance of the best 14 methods is 44.2%, i.e., we get a 21.4% improvement over the older methods. The sequence of these 14 most-contributing methods is shown in Table 10.

There are several issues related to the previous evaluations that need explanation. First, the final 14-method  performance reported in (Kaliszyk and Urban, 2014) was 39%, while here it is only 36.4%. The 39% were measured on the whole older version of Flyspeck, while the 36.4% here is the performance of the old methods limited to the 1796 problems selected from the set of 10,779 theorems that are preserved between the old and the new version of Flyspeck. Additionally, we have recently reported (Kaliszyk and Urban, 2013e) an improvement of the 39% performance to 47% by using better learning methods and better E strategies. However, that preliminary evaluation has been so far done only on a smaller random subset of the old Flyspeck, so we do not yet have the corresponding data for all the 10,779 preserved theorems and their 1796-big subselection used here for the comparison. A very rough extrapolation is that the 47% performance on the smaller subset will drop to 45% on the whole old Flyspeck, which when proportionally decreased by the performance decrease of the old methods (39/36.4) yields 42% performance estimate on the new 1796-big set. Third, we should note that the new lemma-based methods are so far based only on learning from the ITP (human-proof) dependencies, which is for Flyspeck quite inferior to learning on the dependencies extracted from minimized ATP proofs of the problems. Fourth, we do use here the (one) best predictor and the ATP strategies developed in (Kaliszyk and Urban, 2013e), however, we have not so far explored and globally optimized as many parameters (learners, features and their weightings, premise slices, and ATP strategies) as we have done for the older non-lemma methods; such global optimization is future work.

So while the 21.4% improvement over (Kaliszyk and Urban, 2014) is valid, a full-scale evaluation of all the methods on the whole new Flyspeck^9^ will likely show a smaller improvement due to the lemma-mining methods. A very conservative estimate is again 5% (44.2%/42%), however a much more realistic is probably 10%, because the effect of learning from ATP proofs is quite significant. Higher lemma-based performance on Flyspeck than on the core HOL Light is quite plausible: the core HOL Light library is much smaller, more stable and optimized, while Flyspeck is a fast-moving project written by several authors, and the library structuring there is more challenging.

As expected the graph cutting method (*MC*) does indeed produce the smallest dependency graph passed to the predictors. For 10,000 added lemmas the average number of edges in the *MC*-produced dependency graph is 37.0, compared with the average over all strategies being 42.9 dependencies per theorem and epcllemma producing graphs with the biggest number: 63.1 dependencies. This however does not yet correspond to high success rates in the evaluation, possibly due to the fact that graph cutting does not so far take into account the number of small steps needed to prove the added lemma. On the other hand, Table 10 shows that graph cutting provides the most complementary method, adding about 25% more new solutions to the best method available.

Finally we analyze the influence of the number of added lemmas on the success rate in Table 11. As expected adding more lemmas does improve the general performance up to a certain point. The experiments performed with all the lemmas added are already the weakest. However, when it comes to the problems solved only with a certain number of lemmas added, using all the lemmas comes out complementary to the other numbers.

### 7.3 Examples

We have briefly looked at some first examples of the problems that can be solved only with the lemma-based methods. So far we have detected two main effects how such new proofs are achieved: (i) the new lemma (or lemmas) is an easy-but-important specialization of a general theorem or theory, either directing the proof search better than its parents or just working better with the other premises, and (ii) no new lemma is needed, but learning on the newly added lemmas improves the predicting systems, which then produce better advice for a previously unsolvable problem. The second effect is however hard to exemplify, since the number of alternative predictions we tried is high, and it usually is not clear why a particular prediction did not succeed. An example in the first category is the theorem

AFFINE_ALT:⊢affines⟺(∀xyu.xINs∧yINs⟹(&1−u)%

which E can prove using 15 premises, three of them being new lemmas that are quite “trivial” consequences of more general theorems:

NEWDEP309638:⊢&1−a+a=&1

NEWDEP310357:⊢−&1⁎−&1=&1

NEWDEP272099_conjunct1:⊢∀m.&m+−&m=&0

Another example in the first category is theorem

MEASURABLE_ON_NEG:⊢∀fs.measurable_onfs⟹measurable_on(\x.−fx)s

whose proof uses a few basic vector facts plus one added lemma:

NEWDEP1643063:measurable_onfs⊢measurable_on((%

This lemma appeared in the proof of the close theorem

MEASURABLE_ON_CMUL:⊢∀cfs.measurable_onfs⟹measurable_on(\x.c%

The lemma here is almost the same as the theorem where it was first used, but it likely works better in the FOF encoding because the lambda function is eliminated.

## 8 Future work and conclusion

We have proposed, implemented and evaluated several approaches that try to efficiently find the best lemmas and re-organize a large corpus of computer-understandable human mathematical ideas, using the millions of logical dependencies between the corpus' atomic elements. We believe that such conceptual re-organization is a very interesting AI topic that is best studied in the context of large, fully semantic corpora such as HOL Light and Flyspeck. The byproduct of this work are the exporting and post-processing techniques resulting in the publicly available proof graphs that can serve as a basis for further research.

The most conservative improvement in the strength of automated reasoning obtained so far over the core HOL Light thanks to lemma mining is about 5%. The improvement in the strength of automated reasoning obtained over Flyspeck problems is 21.4% in comparison to the methods developed in (Kaliszyk and Urban, 2014), however this improvement is not only due to the lemma-mining methods, but also due to some of the learning and strategy improvements introduced in (Kaliszyk and Urban, 2013e). A further large-scale evaluation using learning from ATP proofs and global parameter optimization is needed to exactly measure the contribution and overall strength of the various AI/ATP methods over the whole Flyspeck corpus.

There are many further directions for this work. The lemma-mining methods can be made faster and more incremental, so that the lemma quality is not completely recomputed after a lemma is named. Fast PageRank-based clustering should be efficiently implemented and possibly combined with the other methods used. ATP-style normalizations such as subsumption need to be correctly merged with the detailed level of inferences used by the HOL Light proof graph. The existing ITP proof-reconstruction methods (Smolka and Blanchette, 2013; Kaliszyk and Urban, 2013d) will need to be updated to handle not just the top-level theorems, but also the intermediate lemmas. Guessing of good intermediate lemmas for proving harder theorems is an obvious next step, the value of which has already been established to a certain extent in this work.

## Figures and Tables

**Algorithm 1:**
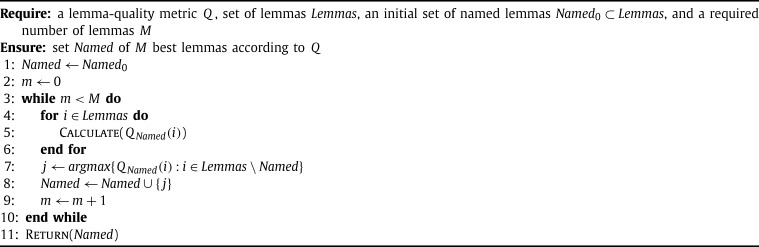
Best lemmas.

**Table 1:** The top-level available data and processing statistics of the analyzed corpora.

	HOL Light (179)	Flyspeck (3437)
Named theorems	2239	23,323
Distinct named conjuncts	2542	24,745
Constant definitions	234	2106
Type definitions	18	29
Processing time	2 m 09 s	327 m 56 s
Processing memory	214 MB	1645 MB

**Table 2:** The sizes of the inference graphs.

	HOL Light graph		Flyspeck graph	
	nodes	edges	nodes	edges
kernel inferences	8,919,976	10,331,922	1,728,861,441	1,953,406,411
TRACE0	2,435,875	3,476,767	206,699,009	302,799,816
TRACE1	2,076,682	3,002,990	159,102,636	233,488,673
tactical inferences	148,514	594,056	11,824,052	42,296,208
tactical trace	22,284	89,981	1,067,107	4,268,428

**Table 3:** Comparison of the methods evaluated on the kernel traces on the 1954 HOL Light theorems.

Strategy	Theorems (%)	Unique	Theorems
$Q_{1\ldots 3}$ (direct quality, Section 5.1)	62.897	68	1229
$PR_{1\ldots 5}$ (PageRank, Section 5.3)	58.700	17	1147
$EQ_{1\ldots 2}$ (epcllemma, Section 5.2)	57.011	4	1114
$MC_{1\ldots 2}$ (graph cut, Section 5.4)	47.288	1	924
total	64.125		1253

only named	54.452	0	1064
total	64.125		1253

(14 best)	63.050	92	1232
combined 14 best	66.172		1293
total	68.833		1345

**Table 4:** Success rate depending on kind of trace used and depending on the number of added theorems.

Added theorems	Success rate	Unique	Thms
TRACE2	62.078	48	1213
TRACE0	59.365	12	1160
TRACE1	58.802	17	1149

10,000	63.562	138	1242
1000	55.374	9	1082

**Table 5:** Success rate of $Q_{i}$ depending on the quality formula.

Lemma quality	Success rate	Unique	Thms
$Q_{1}$ ($\frac{U(i)⁎D(i)}{S(i)}$)	58.751	21	1148
$Q_{2}$ ($\frac{U(i)⁎D(i)}{S{\left(i\right)}^{2}}$)	57.932	10	1132
$Q_{3}^{1.1}$ ($\frac{U(i)⁎D(i)}{1.1^{S(i)}}$)	57.523	8	1124
$Q_{3}^{1.25}$ ($\frac{U(i)⁎D(i)}{1.25^{S(i)}}$)	53.685	2	1049
$Q_{3}^{1.05}$ ($\frac{U(i)⁎D(i)}{1.05^{S(i)}}$)	52.866	0	1033
$Q_{2}^{2}$ ($\frac{U(i)}{S(i)}$)	52.456	4	1025
$Q_{3}^{1.025}$ ($\frac{U(i)⁎D(i)}{1.025^{S(i)}}$)	49.437	0	966
$Q_{1}^{2}$ ($\frac{U{\left(i\right)}^{2}}{S(i)}$)	49.437	8	966
$Q_{1}^{0}$ ($\frac{D{\left(i\right)}^{2}}{S(i)}$)	46.469	3	908
$Q_{2}^{0}$ ($\frac{D(i)}{S(i)}$)	44.882	1	877

**Table 6:** Success rate of epcllemma depending on kinds of formulas given.

Added theorems	Success rate	Unique	Thms
Preserve size	55.732	15	1089
Preserve size and initialize	55.322	8	1081
MESON translation	47.339	11	925

**Table 7:** Success rate of PageRank depending on kinds of formulas given.

Added theorems	Success rate	Unique	Thms
$PR_{6}$ ($\frac{PR_{1}(i)+PR_{3}(i)}{S(i)}$)	53.173	22	1039
$PR_{3}$ (reverse $PR_{1}$)	52.968	13	1035
$PR_{5}$ ($PR_{1}(i)+PR_{3}(i)$)	52.252	14	1021
$PR_{2}$ ($\frac{PR_{1}(i)}{S(i)}$)	46.008	5	899
$PR_{4}$ ($\frac{PR_{3}(i)}{S(i)}$)	45.650	8	892
$PR_{1}$	42.272	1	826

**Table 8:** Theorems found with chains of given lengths.

Length of chains	Success rate	Unique	Thms
–	54.5	519	1066
1	32.0	75	627
2	12.2	30	239
3	2.3	12	46
4	1.1	4	22
5	0.3	4	6
6	0.3	4	6
>6	0.1	2	2
Total	64.6		1264

**Table 9:** Comparison of the methods evaluated on the tactical trace and the 1796 Flyspeck theorems.

Strategy	Theorems (%)	Unique	Theorems
$PR_{1\ldots 5}$ (pagerank, Section 5.3)	36.860	39	662
$Q_{1\ldots 3}$ (direct quality, Section 5.1)	35.913	31	645
$MC_{1\ldots 2}$ (graph cut, Section 5.4)	30.178	1	542
$EQ_{1\ldots 2}$ (epcllemma, Section 5.2)	29.677	0	533
all lemmas	21.047	26	378
only named	28.786	1	517
14 best	37.584		675
total	40.813		733

(14 best)	36.414	127	654
combined 14 best	44.209		794
total	47.884		860

**Table 10:** Combined 14 best covering sequence.

Strategy	Pred.	Feat.	Lemmas	Prem.	ATP	Success	Thms
	NBayes	typed, notriv	ATP-deps	154	epar	24.666	443
$MC_{2}$	*k*-NN	typed	1000 lemmas	128	epar	31.180	560
All lemmas	*k*-NN	types	all lemmas	32	z3	34.855	626
	NBayes	types, notriv	ATP-deps	1024	epar	36.693	659
$Q_{1}$	*k*-NN	types	60,000 lemmas	32	z3	38.474	691
	NBayes	typed, notriv	ATP-deps	92	vam	40.033	719
Only Named	*k*-NN	types	-	512	epar	40.980	736
$Q_{1}^{2}$	*k*-NN	types	60,000 lemmas	32	z3	41.759	750
	*k*-NN160	types, notriv	ATP deps	512	z3	42.316	760
$Q_{1}^{0}$	*k*-NN	types	60,000 lemmas	32	z3	42.762	768
$PR_{6}$	*k*-NN	types	20,000 lemmas	512	epar	43.207	776
	NBayes	fixed	Human deps	512	epar	43.541	782
$PR_{1}$	*k*-NN	types	20,000 lemmas	32	z3	43.875	788
$PR_{4}$	*k*-NN	types	20,000 lemmas	128	epar	44.209	794

**Table 11:** Influence of the number of added lemmas on the success rate.

Added lemmas	Theorems (%)	Unique	Theorems
60,000	36.804	37	661
20,000	35.523	18	638
10,000	33.463	3	601
0	28.786	1	517
5000	27.951	0	502
1000	27.895	0	501
all	21.047	26	378

## Footnotes

1 This paper is an extended version of (Kaliszyk and Urban, 2013b).

2 23,323 theorems are in the HOL Light/Flyspeck library (SVN revision 3437), about 20,000 are in the Isabelle/HOL library, and about 50,000 theorems are in the Mizar library.

3 These experiments were done on a earlier version of Flyspeck (SVN revision 2887) than is used here (SVN revision 3437), where the number of theorems is 23,323.

4 A similar scenario has been introduced in 2013 also for the LTB (Large-Theory Batch) division of the CASC competition.

5 This behavior obviously depends on the premise-selection algorithm. It is likely to occur when the premise selection is mainly based on symbolic similarity of the premises to the conjecture. It is less likely to occur when complementary semantic selection criteria are additionally used as, e.g., in SRASS (Sutcliffe and Puzis, 2007) and MaLARea (Urban et al., 2008).

6 In particular, several initial experiments done so far with Veroff's hints over the MPTPChallenge and MPTP2078 benchmarks were so far unsuccessful.

7 http://cl-informatik.uibk.ac.at/~cek/lemma_mining/.

8 The possibility to create conjunctions is quite a significant difference to the clausal setting handled by the existing tools. A longer clause is typically weaker, while longer conjunctions are stronger. A dependence on a longer conjunction should ideally be treated by the evaluating heuristics as a dependence on the multiple conjuncts. Note that for the tactical trace we already split all conjunctions in the trace.

9 Such evaluation could take another month with our current resources.
